# Manzamine-A: Unraveling the Chemical and Biological Tapestry of a Marine-Derived Drug Lead

**DOI:** 10.3390/md24060190

**Published:** 2026-05-26

**Authors:** Xuan Wang, Hengbo Wang, Yuansai Kang, Xiaojing Tang, Linlin Ma

**Affiliations:** Department of Pharmacology, School of Basic Medicine, Qingdao University, Qingdao 266071, China; 2025021805@qdu.edu.cn (H.W.); kangyuansai@qdu.edu.cn (Y.K.); tangxiaojing@qdu.edu.cn (X.T.); malinlin@qdu.edu.cn (L.M.)

**Keywords:** manzamine-A, microbial source, biological activity, pharmacokinetics, chemical synthesis, the derivatives and analogues

## Abstract

Manzamine-A (MA), a complex β-carboline alkaloid isolated from various genera of marine sponges, has attracted significant attention due to its unique structure and broad spectrum of potent biological activities. Despite the therapeutic potential, its development is limited by challenging natural supply and suboptimal pharmacokinetics. To address these barriers, innovative total syntheses of its intricate polycyclic framework have been achieved, enabling the development of semi-synthetic and synthetic analogues aimed at improving potency and drug-like properties. This review comprehensively outlines the progress in understanding this marine natural product, mainly focusing on its microbial origin, biological activities, pharmacokinetic behavior, chemical synthesis, and derivatives’ and analogues’ development. By integrating these diverse yet interconnected fields of research, this review bridges the critical gap between the natural product’s discovery and its clinical translation. Additionally, it also provides a roadmap for future drug development, highlighting how interdisciplinary collaboration can unlock the therapeutic potential of MA as a viable clinical candidate.

## 1. Introduction

Historically, the vast majority of new drugs are generated from natural products, having been proven to be a rich source of compounds for drug discovery [1]. Natural products (namely secondary metabolites), in a broad sense, usually refer to the components or metabolites of animals, plants, insects, microorganisms and marine organisms, etc., mainly including flavonoids, peptides, alkaloids, polysaccharides, glycosides, polyphenols, terpenoids, quinones, etc. [2,3,4,5,6] and playing a very important role in the treatment of many diseases [7,8,9,10,11]. According to statistics, 65.3% of drugs belong to natural products or their derivatives among the 1211 new chemical entities approved worldwide from 1981 to 2014, especially in the fields of antibiotics, immunosuppressants, anti-tumor drugs, and lipid-lowering drugs [12,13,14,15,16]. From 2012 to 2016, the number of new drugs approved by the FDA decreased year by year, but the number of natural product-related drugs remained stable [12]. Even now, natural products are still an important source of new drugs.

The ocean is home to about 80 percent of all plants and animals on Earth, as well as about 1500 species of bacteria, and it is therefore a treasure trove of biological active natural products [17]. Marine natural products have become the main sources for the discovery of important lead compounds and innovative drugs due to their unique properties and novel structures, and marine resources have attracted increasing attention from biomedical researchers [18,19]. Up to now, more than 32,000 new compounds of marine origin have been discovered. Currently, modern technologies make it possible to attain unexplored sea depths, making marine biota available to researchers [20,21]. Therefore, more and more lead compounds and innovative drugs are gradually being discovered from the treasure trove of the ocean.

Manzamine-A (MA) is a member of manzamine alkaloids with complex and special structure extracted from marine animal sponges and is a landmark molecule in marine natural product chemistry [22]. Its complex pentacyclic framework, characterized by a fused and bridged ring system incorporating a β-carboline moiety, presents a formidable challenge to synthetic chemists and offers a distinctive scaffold for drug discovery. Beyond its architectural novelty, MA exhibits a remarkable and diverse range of bioactivities including antimalarial, antibacterial, antiviral, anti-inflammatory and anti-atherosclerosis activities, showing great potential in the treatment of various diseases [23,24,25,26]. Accumulating evidence also shows that MA has anti-tumor activity in multiple tumor types, becoming one of the most attractive marine natural products at present [27,28,29,30]. However, the journey from marine sponge to clinical application has been hindered by its “supply problem”—the minute and variable yields from wild-harvested sponge populations. Furthermore, recent studies have shown that long-term or high-dose use of MA may interfere with the bone remodeling process, raising concerns about potential adverse skeletal side effects that require careful evaluation in future clinical safety assessments [31,32].

Despite these challenges, MA stands out among the large manzamine alkaloid family as an exceptionally promising drug discovery seed. Unlike many family members that remain at the stage of preliminary screening, MA has been the subject of the most systematic structure–activity relationship (SAR) studies; key modification sites (e.g., C-8, C-12, and the imine nitrogen) have been extensively explored, yielding clear SAR patterns that are directly instructive for medicinal chemistry optimization. Its architecturally complex skeleton has served as a benchmark for total synthesis over the past three decades, and its in vivo antimalarial efficacy has advanced it into more detailed preclinical evaluation. For these reasons, this review focuses exclusively on MA, providing a comprehensive integration of the multifaceted research surrounding this remarkable alkaloid. We will systematically dissect its journey, beginning with its microbial source and ecological context, then detail its extensive bioactivities across therapeutic domains, followed by an analysis of its pharmacokinetic behavior and the limitations therein. The monumental efforts in chemical synthesis will be reviewed, highlighting the strategic breakthroughs. Finally, we will survey the landscape of derivatives and analogues, discussing how medicinal chemistry has been employed to optimize this natural scaffold. By weaving together these diverse threads, this review seeks to elucidate both the immense potential of MA as a drug lead and the concrete scientific strategies required to translate its complex tapestry into a tangible clinical reality.

## 2. Section Snippets

### 2.1. Microbial Source

Marine sponges are the most primitive and simplest multicellular animals on earth, widely distributed in the world’s oceans, from the shallow sea to the deep sea [33]. Sponges have been living in the ocean for 600 million years and have developed to more than 10,000 species, accounting for 1/15 of the marine animal species [34,35,36]. Biologically, sponges belong to the phylum porifera [37,38,39]. As the name suggests, they have numerous small holes in their bodies, forming a complex “canal system” [40,41,42]. Sponges feed on these small holes to filter plankton and oxygen out of the water and to remove waste [43,44,45]. Although sponges may seem mundane, they play a very important role in marine ecosystems [46]. As a filter, sponges can help clean up harmful substances and microorganisms in seawater and maintain a healthy and clean marine environment [47,48,49]. In addition, sponges are an important food source for many marine organisms, providing rich nutrients to the marine food chain [50,51]. Meanwhile, sponges contribute to approximately 30% of all marine natural products discovered to date [52]. Since they do not have an innate immune system or refined defense structures, they persevere by producing metabolites that act as a self-defense device that allows them to adapt to the most diverse environments of the evolutionary scale and hinder predators [53]. Nowadays, the biological potential of these chemicals and metabolites has been extended to biomedical sciences with multiple effects in molecular and cellular events [54,55,56]. Manzamine-A (MA) is just a natural product derived from marine sponges [25,57].

**Figure 1:**
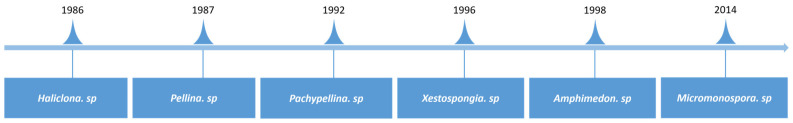
Timeline of key discoveries of MA microbial sources.

The quest to understand the true biosynthetic origin of the potent marine alkaloid MA is a compelling narrative that spans nearly three decades, driven by the need for a sustainable supply of this promising drug lead. MA was first isolated and identified by Higa and his colleagues in 1986 from the marine sponge *Haliclona* sp. collected off Cape Manzamo, Okinawa, Japan, which established the initial biological source of this compound [58]. Then, a dark brown sponge *Pachypellina* sp. was collected from Manado, Sulawesi, Indonesia, and MA was extracted in 1992. Subsequently, MA also has been found in other genera of marine sponges, including *Pellina*, *Pachypellina*, *Xestospongia*, and *Amphimedon* [59,60,61]. However, in 2014, Waters et al. first reported the sponge-associated bacterium (*Micromonospora* sp. M42) isolated from the Indo-Pacific sponge *Acanthostrongylophora* ingens. It was identified and verified as the producer of MA, indicating MA is not produced by the sponges themselves but by their associated microbial symbionts bacteria *micromonospora* sp. M42 [57]. The timeline of key discoveries in research of MA microbial sources is shown in Figure 1.

### 2.2. Biological Activities

#### 2.2.1. Anti-Microbial Activity

The groundbreaking work by Ang et al. in 2000 first demonstrated the potent in vivo antimalarial activity of Manzamine-A (MA) against *Plasmodium berghei* (*P. berghei*) [23]. A single intraperitoneal or oral dose effectively suppressed over 90% of asexual erythrocytic phases of *P. berghei*, significantly prolonging survival in infected mice and far exceeding that of chloroquine and artemisinin [62,63], two of the most important, classic and commonly used antimalarial drugs. Morphological degeneration of parasites observed within 24 h post treatment resemble those reported for chloroquine treatment. Subsequently, Ang et al. further revealed that the efficacy of MA involves immunomodulation, and the MA treatment of infected mice induced an immune response, shifting the host response from Th1-mediated cellular immunity (characterized by IFN-γ production, a key cytokine in Th1 response) to Th2-mediated humoral immunity (characterized by IL-10 production, a key cytokine in Th2 response), which facilitated the eventual parasite clearance in recurrent infected mice [64]. In vitro, MA shows promising activity against both chloroquine-sensitive (D6 clonal cell lines) and -resistant (W2 and W3 clonal cell lines) *Plasmodium falciparum* (*P. falciparum*) strains, highlighting its potential as a novel option for the treatment of drug-resistant malaria [65]. Additionally, MA exhibits potent activity against *Leishmania donovani* in vitro, surpassing the standard antileishmanial drug pentamidine [66]. These reports confirm that MA has a significant antiparasitic properties, and the corresponding IC50 values for the drugs listed above are provided in Table 1.

Furthermore, MA exhibits a notable broad-spectrum antibacterial profile, with particular efficacy against Gram-positive and mycobacterial strains. Initial studies established MA’s inhibitory activity against common Gram-positive bacteria Bacillus subtilis and *Staphylococcus aureus* (*S. aureus*) [60,65]. However, it is crucial to note that much of the potent and specific anti-methicillin-resistant *S. aureus* (MRSA) activity has been documented for synthetic analogues and derivatives of the manzamine core structure, rather than the parent MA itself [67]. Simithy et al. identified MA as a potent inhibitor of shikimate kinase from Mycobacterium tuberculosis (MtSK), a promising drug target, thereby establishing its direct anti-tubercular mechanism and candidacy for therapeutic development [68]. Beyond Mycobacterium tuberculosis, it exhibits superior potency against *Mycobacterium intracellulare* compared to the clinical antibiotic ciprofloxacin, as reported by Winkler [67,69]. The antibacterial mechanism is under investigation but is distinct from its antimalarial action. Evidence suggests manzamine-based compounds can act as potent synergists with aminoglycoside antibiotics. They are proposed to bind to the bacterial ribosomal decoding A-site, disrupting protein synthesis and amplifying the bactericidal effect of drugs like paromomycin [70]. The antifungal activity of MA is evident and more selective but less extensively documented than its antibacterial effects. MA shows critical specificity against certain resilient pathogens including *Cryptococcus neoformans* (*C. neoformans*) and Candida albicans [71,72], highlighting its potential against multiple fungal infections. The IC50 values of all the drugs above are shown in Table 2 and Table 3.

MA also has a broad-spectrum antiviral profile. Against herpes simplex virus type 1 (HSV-1), MA effectively inhibited viral replication and infectious virus yield in corneal cells, exhibiting activity superior to acyclovir [73], the first-choice drug for HSV-1 infection. Mechanistic studies have suggested it modulates viral gene expression (e.g., ICP0) and virion host shutoff activity, indicating the potential for treating HSV-1 ocular infections [74]. In addition, MA in vitro shows potent activity against human immunodeficiency virus type 1 (HIV-1). Although its potency does not surpass that of the first-line drug zidovudine, these findings underscore MA’s core structure as a promising scaffold for developing novel antiviral agents [75]. The IC50/EC50 values of all the drugs above are presented in Table 4.

#### 2.2.2. Anti-Neurodegenerative Activity

Marine natural products represent a promising source for modulating neuroinflammation, a key process in neurodegenerative diseases. Among them, manzamine alkaloids demonstrate significant activity in regulating critical neuroinflammatory mediators. Pomponi et al. provided the first experimental evidence that MA, at non-toxic in vitro concentration, potently inhibits the release of key neuroinflammatory mediators (thromboxane B_2_/TXB_2_ and superoxide anion/O_2_^−^) from activated microglia, particularly against PMA-stimulated pathways [76]. Based on its potency and selectivity, MA is highlighted as a promising lead candidate for developing novel therapeutics targeting neuroinflammatory components of neurodegenerative diseases. Beyond immunomodulation, MA has been identified as a novel inhibitor of glycogen synthase kinase-3β (GSK-3β). It effectively reduces pathological tau hyperphosphorylation in human neuronal cells by selectively inhibiting GSK-3β and CDK-5, two central kinases in Alzheimer’s disease pathogenesis [77]. This positions MA not only as a lead compound for neuroinflammatory conditions but also as a valuable scaffold for the rational design of targeted therapies against Alzheimer’s disease.

#### 2.2.3. Anti-Atherosclerosis Activity

In 2013, Eguchi et al. first reported a pivotal finding: MA also exhibits promising activity against cardiovascular metabolic disorders [26]. The study showed that MA inhibited acetylated LDL-induced foam cell formation in human macrophages and suppressed cholesterol ester synthesis by directly inhibiting acyl-CoA cholesterol acyltransferase (ACAT) activity. When administered orally to apolipoprotein E (apoE)-deficient mice, MA significantly mitigated dyslipidemia, reducing serum levels of total cholesterol, LDL-cholesterol, and triglycerides, and it substantially decreased the atherosclerotic lesion area in the aortic sinus, demonstrating its potent anti-atherosclerotic effects both in vitro and in vivo. These collective findings make MA a promising lead compound for the prevention or treatment of hyperlipidemia and atherosclerosis.

#### 2.2.4. Anti-Tumor Activity

Cumulative evidence collected in recent decades suggests that MA also exhibits anti-tumor activity in several types of malignancies [78], such as pancreatic, colorectal, cervical, prostatic, breast cancers and glioblastoma, as well as in benign tumors like uterine fibroids.

In 2011, Guzmán et al. [79,80] verified that MA inhibits key oncogenic processes, including clonogenic survival, migration, and invasion, while sensitizing cells to TRAIL-induced apoptosis in pancreatic cancer AsPC-1 cells. Mechanistically, MA was further identified by Guzmán et al. [28] as an uncoupler of vacuolar ATPase (v-ATPase), namely, a proton pump responsible for pumping H+ in the cytoplasm across the membrane into the lysosome. MA directly impaired the function of this essential element of lysosomal acidification and subsequent degradative capacity. By inhibiting the activity of v-ATPase, MA disrupts autophagic flux in pancreatic cancer cells, as evidenced by the accumulation of LC3-II and p62/SQSTM1, by phenocopying the specific lysosomal inhibitor bafilomycin A1, revealing a unique mechanism that contributes to its anticancer effects and indicates that MA is a promising therapeutic strategy for targeting autophagy in pancreatic cancer.

MA also has significant therapeutic potential against colorectal cancer (CRC) through direct cytotoxic mechanisms and computational discovery. In vitro, Li et al. [27] reported that MA inhibits proliferation, induces G_0_/G_1_ cell cycle arrest via the p53/p21/p27 axis and triggers mitochondrial apoptosis in CRC cell lines. It also suppresses metastasis by inhibiting the epithelial–mesenchymal transition (EMT) process in HCT116 cells, evidenced by the downregulation of Snail, Slug, Twist and upregulation of E-cadherin. Moreover, MA has been recurrently identified as a top-ranked therapeutic candidate via independent bioinformatics and molecular docking studies. These studies pinpointed MA as a high-affinity ligand for critical targets, including CRC-related core genes (e.g., AURKA, TOP2A) and the tumor-specific antigen LY6G6D, validating its potential for early-stage intervention and targeted therapy [81,82,83]. This convergence of experimental and computational evidence robustly presents MA as a promising multi-target agent for CRC treatment.

Additionally, MA has been confirmed to be an effective candidate compound for the prevention and treatment of cervical cancer. Karan et al. [29] verified that MA exerts potent antiproliferative effects at relatively low and non-cytotoxic concentrations on cervical cancer cells (C33A, HeLa, SiHa, and CaSki) in vitro; prevents cell cycle progression via the regulation of p53/p21 pathway in SiHa and CaSki cells; and decreases the oncogenesis-associated oncoprotein SIX1 expression in cervical cancer. Furthermore, a vital study conducted by Mayer et al. [84] identified MA as a novel, potent, and selective inhibitor of the ribosomal S6 kinase (RSK), a vertebrate family of cytosolic serine-threonine kinases that act downstream of the RAS/ERK1/2 pathway, which phosphorylates substrates shown to regulate several cellular processes, including growth, survival, and proliferation. MA exhibits a 10-fold selectivity for RSK1 (IC50: 15.01 µM) over its isoform RSK2 (IC50: 108.4 μM), as verified via the inhibition of proteins expression of RSK1 and RSK2 by MA in SiHa and CaSki human cervical carcinoma cell lines and also supported by computational docking experiments, which revealed stronger binding interactions with the RSK1 N- and C-terminal domains. This discovery confirmed that RSK1 might be a unique molecular target of MA in cervical cancer treatment.

Han et al. [30,85] also validated the significant anticancer activity of MA in breast cancer, time- and dose-dependently suppressing the proliferation, migration, and invasion of MDA-MB-231 and MCF-7 cells. Mechanistically, MA induces secretory autophagy by promoting autophagosome formation while blocking their lysosomal degradation via regulating the RIP1/AKT/mTOR signaling pathway, as characterized by the potent accumulation of autophagy-related proteins within secreted exosomes, an effect synergistically enhanced by the lysosomotropic agent chloroquine. These findings highlight the role of MA as a modulator of autophagic flux and suggest that autophagic exosomes may serve as valuable biomarkers for monitoring lysosomal function and the efficacy of lysosome-targeting therapies in breast cancer [85].

Karan et al. [86] found that MA has significant anti-tumor efficacy against castration-resistant prostate cancer (CRPC). It effectively inhibits various prostate cancer cells’ growth in vitro and suppresses xenograft tumor growth in vivo. MA mechanistically orchestrates a multi-faceted blockade of the androgen receptor (AR) pathway, a cornerstone of CRPC progression. It downregulates the expression of both full-length AR (AR-FL) and the therapy-resistant splice variant AR-V7, concurrently reducing the levels of key AR-regulated genes, including prostate-specific antigen (PSA). This comprehensive suppression is achieved by MA’s unique action of inhibiting the transcriptional regulator E2F8, thereby disrupting its binding to DNA and ultimately repressing AR gene transcription and synthesis. This targeted intervention presents MA as a promising therapeutic strategy through which to overcome variant-driven resistance in advanced prostate cancer.

Taneja et al. [87] identified MA as an effective inhibitor of glioblastoma (GBM) cell growth by specifically targeting and inhibiting the activating tyrosine phosphorylation of GSK3β kinase. This inhibition leads to the downregulation of key oncogenic splicing factors, including hnRNPA1 and SF2/ASF, which are crucial for generating pro-survival alternative splice variants. Consequently, MA treatment reduces the expression of anti-apoptotic regulators (such as MCL1 and Survivin), restores the expression of the tumor suppressor Anxa7, and ultimately suppresses glioma cell viability and tumorigenic colony formation, highlighting its potential as a multi-faceted therapeutic agent in GBM.

Huang et al. [88] demonstrated that MA exerts potent anti-tumor effects against uterine leiomyomas by targeting sterol O-acyltransferase (SOAT), a key enzyme in cholesterol metabolism. Through SOAT inhibition, MA suppresses tumor growth via a dual-pronged mechanism; it disrupts the fibrotic tumor microenvironment by blocking the SOAT/β-catenin pathway and related extracellular matrix (ECM) deposition while simultaneously inducing lethal oxidative and endoplasmic reticulum stress that triggers apoptotic cell death. This integrated action on both the fibroid tissue structure and the survival of tumor cells highlights MA’s novel mechanism and its promising therapeutic potential for a condition with limited medical treatment options. The anti-tumor effects and mechanisms of MA in the above tumor types are summarized in Table 5.

#### 2.2.5. Anti-Bone Remodeling Activity

It should be noted that recent studies have revealed that MA modulates bone remodeling through its dual effects on both bone-forming and bone-resorbing cells. Cray et al. [31,32] indicated MA exerted inhibitory effects on both bone formation in osteoblasts and bone resorption in osteoclasts in vitro by modulating the expression of SIX1, a key developmental gene that is crucial for bone development and homeostasis. They exhibited that MA significantly promotes cell apoptosis, decreases cell viability, and diminishes alkaline phosphatase (osteogenic differentiation marker) activity in osteoblasts and their progenitors, showing great sensitivity to MA treatment. Furthermore, MA significantly reduces the viability of preosteoclasts and osteoclasts, induces apoptosis, and inhibits their differentiation into functional osteoclasts. These findings suggest that MA might disrupt the dynamic balance of bone remodeling, affect bone health, and pose potential risks to skeletal development and repair capabilities, especially under prolonged or high-dose exposure.

### 2.3. Pharmacokinetics

MA represents a highly promising marine-derived lead compound for the treatment of multiple diseases, and a thorough understanding of its pharmacokinetic (PK) profile is critical for evaluating its drug-like properties and therapeutic potential. Nevertheless, due to its structural complexity and limited supply, systematic PK studies remain in a relatively early stage, relying primarily on preclinical studies. Existing data reveal the characteristics, challenges, and potential optimization strategies of its PK behavior.

MA presents significant oral bioavailability challenges due to its inherent physicochemical properties and biological interactions. As a complex lipophilic alkaloid, it has poor aqueous solubility, which fundamentally restricts its dissolution and subsequent absorption in the gastrointestinal tract [24]. Data on its oral bioavailability remains limited, with evidence suggesting it is low (20.6%) or highly variable [24]. Preliminary studies in mouse models confirm that bioavailability following intraperitoneal (i.p.) injection is substantially higher than after oral administration, which is why i.p. injection has been the typical route in animal studies [24]. To overcome this solubility limitation, the hydrochloride salt form (MA hydrochloride) has been synthesized and is now the primary form used in most in vivo investigations [24]. MA has exhibited a broad tissue distribution in animal models. Notably, several studies investigating its antimalarial and anti-neuroinflammatory effects indicate that MA can cross the blood–brain barrier (BBB) and achieve therapeutically relevant concentrations in the central nervous system [25]. This is a critical PK foundation for its potential use in treating cerebral malaria, Alzheimer’s disease-related targets (e.g., GSK-3β), or CNS viral infections [75,77]. In addition, MA is predicted to have a high binding affinity to plasma proteins, such as albumin. While this aids in its solubilization and transport in the bloodstream, high protein binding reduces the concentration of free pharmacologically active drugs and can influence its volume of distribution and clearance. Quantitative data on its exact protein binding percentage are pending. The metabolism of MA is a central area of uncertainty in its PK profile. There is very limited research identifying the specific metabolic pathways and active/toxic metabolites of MA in vivo. Given its complex polycyclic nitrogen-containing structure, MA is likely metabolized predominantly by hepatic cytochrome P450 (CYP) enzymes, particularly isoforms like CYP3A4 and CYP2D6 [25]. This predicts a potential risk for drug–drug interactions (e.g., when co-administered with strong CYP3A4 inhibitors or inducers). Published data on the excretion pathways of MA are also scarce. Based on its physicochemical properties and the excretion patterns of similar alkaloids, MA and its metabolites are likely eliminated primarily via biliary excretion into feces, with a fraction excreted renally in urine [71]. Limited animal study data (e.g., in rats) have indicated MA to have low metabolic clearance and a reasonably long elimination half-life in plasma, although the exact value varies with the route of administration, dose, and species [25]. No human half-life data are available.

### 2.4. Chemical Synthesis

**Figure 2:**
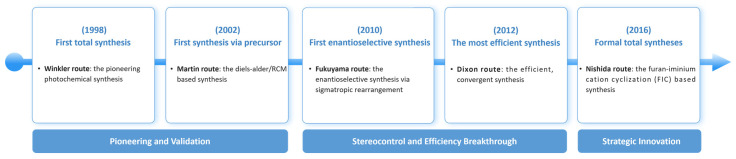
Timeline of MA total synthesis.

MA is a structurally formidable marine alkaloid characterized by a unique pentacyclic framework integrating a planar β-carboline (pyrido [3,4-b]indole) unit with a complex, bridged tetracyclic core [58]. This core consists of fused cyclohexene (A), piperidine (B), and pyrrolidine (C) rings forming a strained [5.5.6]tricyclic system, which is further extended by an eight-membered hexahydroazocine ring (D) and a hallmark thirteen-membered macrocyclic cyclotridecene ring (E), as shown in the graphical abstract. The intriguing structure, with the molecular formula C_36_H_44_N_4_O, exhibits significant stereochemical complexity, featuring multiple stereogenic centers including a fully substituted carbon at the ring junctions, which collectively define its three-dimensional shape and potent biological activities and make it a high-profile target for total synthesis. The synthesis of MA has long stood as the “Mount Everest” in synthetic organic chemistry, a challenge spanning over a decade that mirrors the evolution in synthetic methodology and strategy, highlighting the paradigm shifts from early, linear proofs of concept to modern, efficient, and divergent strategies. The journey encapsulates major advancements in synthetic methodology, including photocycloadditions, pericyclic reactions, ring-closing metathesis (RCM), tandem cyclizations, and novel biomimetic hypotheses. The following are several key phases in the development of MA synthesis. The timeline of MA’s total synthesis is shown in Figure 2.

#### 2.4.1. Early Explorations and Retrosynthetic Analysis (1990s)

Following the structure elucidation of MA, leading synthetic groups engaged in retrosynthetic analysis. The central challenge was the efficient construction of the strained and densely functionalized 13-membered AZA ring (E-ring). Early strategies generally focused on a disconnection into a β-carboline fragment and a highly functionalized hydroisoquinoline fragment, followed by late-stage coupling via amide bond formation or C-N cross-coupling [89]. Although no total synthesis was achieved in this period, it laid the strategic groundwork for subsequent campaigns.

#### 2.4.2. Pioneering Total Syntheses

##### First Total Syntheses: A Photochemical Synthesis (Winkler Route, 1998)

**Figure 3:**
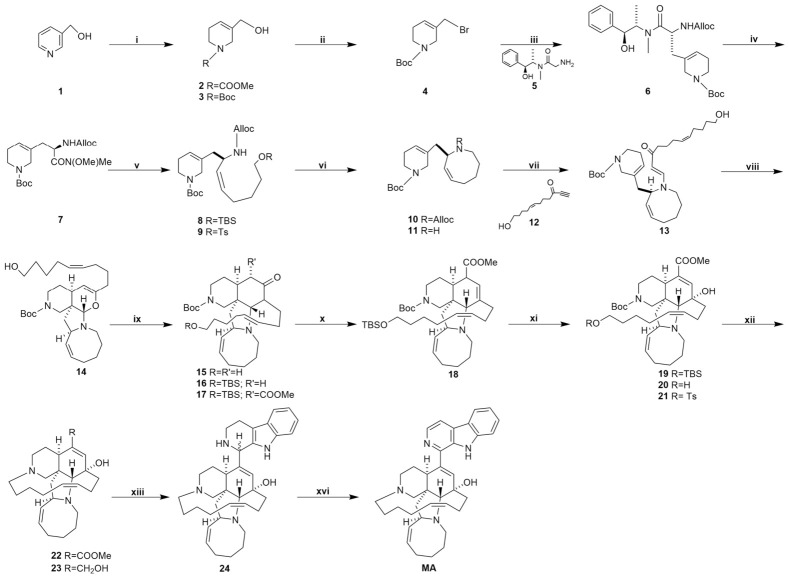
Total synthesis of MA by Winkler and coworkers. Reagents: (i) BnBr; NaBH_4_; MeOCOCI, 50–60%; KOH, aq. MeOH; Boc_2_O, 89%; (5 steps) (ii) Ph_3_P,Br_2_, imidazole, 93%; (iii) LDA, LiCl; 5; AUocCl, 87%; (iv) NaOH, 92%; EtOCOCl, NMM, HN(Me)OMe-HCI, 71%; (v) LAH, 83%; KHMDS, Ph_3_P(CH_2_)_5_OTBSBr, 75%; PPTS, MeOH, 98%; TsCl, 88%; (vi) NaH, 82%; (PPh_3_)_4_Pd°, dimedone, 90%; (vii) 12, 99%; (viii) *hv*; (ix) C_5_H_5_N, AcOH, 20%; TBSCl, 87%; LHMDS, MeOCOCN, 90%; (x) NaBH_4_, 93%; MsCl, Et_3_N, 95%; DBU, benzene, 90%; (xi) *m*-CPAB; NAOMe, 69%; TBAF, 94%; TsCl, TEA, 96%; (xii) TFA, 100%; (Pr)_2_NEt, 12%; (xiii) DIBAl-H, 83%; C_10_H_12_N_2_O, TFA, 58%; (xvi) DDQ, 50%.

The first total synthesis by the group of Prof. Jeffrey D. Winkler [89] at the University of Pennsylvania was a landmark achievement. Its centerpiece was an ingenious tandem [2+2] photocycloaddition/retro-[2+2] fragmentation of a chiral vinylogous amide. This single photochemical transformation directly assembled the advanced tetracyclic core (A, C, D rings and a B-ring precursor), solving the most complex ring-formation problem. Despite its length (37 steps, shown in Figure 3) and low overall yield, this route demonstrated the profound power of pericyclic reactions to generate molecular complexity.

##### First Synthesis via Precursor Ircinal a: The Diels–Alder/RCM-Based Synthesis (Martin Route, 2002)

**Figure 4:**
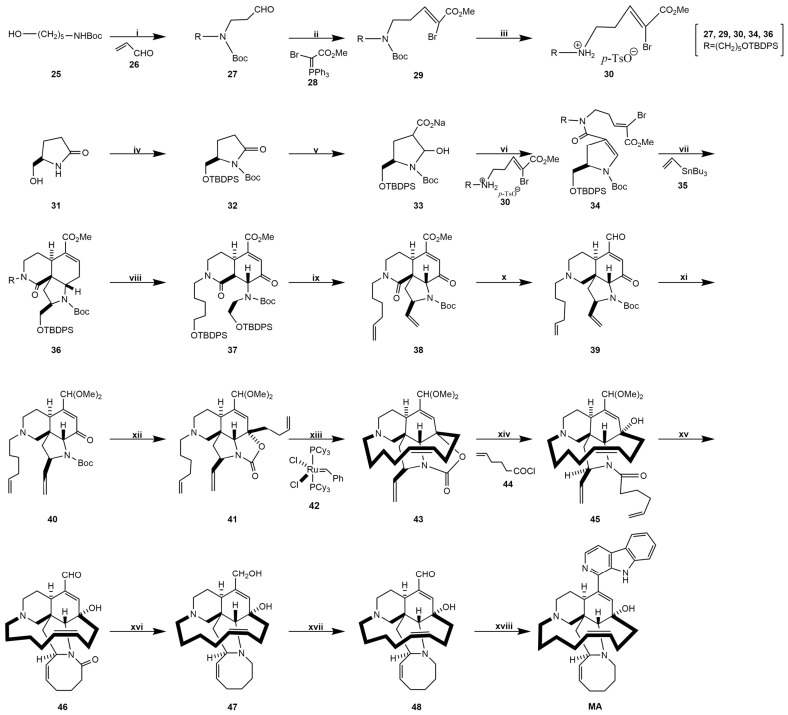
Total synthesis of MA by Martin and coworkers. Reagents: (i) TBDPS-Cl; 26, 71%; (ii) 28, CH_2_Cl_2_, 91%; (iii) TMS-OTf, 2,6-lutidine, *p*-TsOH, 91%; (iv) TBDPS-Cl, imidazole, DMF; (Boc)_2_O, DMAP, Et_3_N, 86%; (v) LHMDS, THF; CO_2_, −78 °C; NABH_4_, EtOH, 0 °C; Na_2_CO_3_, 99%; (vi) 30, (COCl)_2_, NEt_3_, 79%; (vii) 35, toluene, heating, 68%; (viii) CrO_3_, 3,5-Me_2_C_3_H_2_N_2_, CH_2_Cl_2_, −18 °C, 63%; (ix) HCl, MeOH; (COCl)_2_, DMSO, Et_3_N; Ph_3_P=CH_2_, −78 °C to rt, 47%; (x) DIBAL-H; Dess-Martin periodinane, 53%; (xi) HC(OMe)_3_, MeOH, HCl, 84%; (xii) CH_2_=CHCH_2_CH_2_Li, −78 to −20 °C, H_2_O, 65%; (xiii) 42, 67%; (xiv) 44, KOH, MeOH, heating; Et_3_N, CH_2_Cl_2_, 75%; (xv) 42, 1 NHCl, 26%; (xvi) DIBAL-H, 63%; (xvii) Dess-Martin periodinane, 89%; (xviii) tryptamine, CF_3_CO_2_H; DDQ, Et_3_N.

Shortly after, Martin’s team reported a more convergent and streamlined synthesis (23 steps, shown in Figure 4) that first provided access to MA via its biogenetic precursor, ircinal A [90,91]. A pivotal intramolecular Diels–Alder (IMDA) reaction constructed the A/B/C/ring skeleton. This synthesis was instrumental in pioneering the application of ring-closing metathesis (RCM) for constructing the challenging D and E rings, setting a critical precedent for all subsequent syntheses.

#### 2.4.3. The Quest for Stereocontrol and Efficiency

##### First Enantioselective Total Synthesis via Sigmatropic Rearrangement (Fukuyama Route, 2010)

**Figure 5:**
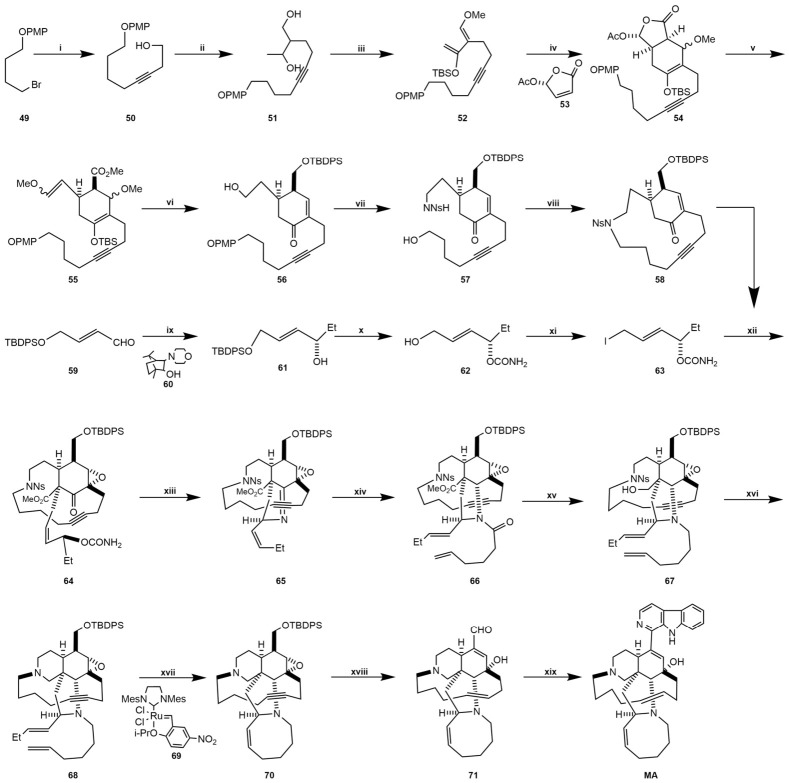
Total synthesis of MA by Fukuyama and coworkers. Reagents: (i) n-BuLi, THPO(CH_2_)_2_CCH, TMEDA, n-Bu_4_NI, THF/HMPA, −78 °C to rt., 91%; CSA, MeOH, rt; 93%; (ii) I_2_, PPh_3_, imidazole, CH_3_CN/Et_2_O, 0 °C to rt; 97%; methyl acetoacetate, NaH, THF, reflux; LiAlH_4_, THF, rt, 91%; (iii) Dess-Martin periodinane, t-BuOH, CH_2_Cl_2_, rt; *p*-TsOH·H_2_O, Na_2_SO_4_, MeOH, rt., 66%; TBSOTf, Et_3_N, Et_2_O, 0 °C; (iv) 53, NaOAc, toluene, MS 3A, reflux, 97% (two steps); (v) Et_3_N, MeOH, rt; evaporation; MeOCH_2_PPh_3_Cl, KHMDS, THF, −78 to 0 °C; MeI, *i*-Pr_2_NEt, DMF, 0 °C, 89%; (vi) LiAlH_4_, Et_2_O, 0 °C, 99%; TBDPSCl, imidazole, CH_2_Cl_2,_ 99%; *p*-TsOH·H_2_O, acetone, 97%; NaBH(OAc)_3_, AcOH, benzene, 40 °C, 88%; (vii) NsNHBoc, DEAD, PPh_3_, benzene, rt, 97%; TFA, rt; evaporation; CAN, MeCN/H_2_O, 0 °C, 81%; (viii) DEAD, PPh_3_, toluene (0.01M), rt, 85%; (ix) 60, Et_2_Zn, hexane/toluene, −10 °C, 75%, 93%; (x) Cl_3_CCONCO, CH_2_Cl_2_, 0 °C; evaporation; Et_3_N, MeOH, rt; evaporation; TBAF, THF, 50 °C; 99%; (xi) MsCl, Et_3_N, CH_2_Cl_2_, 0 °C; NaI, acetone, 50 °C; 51% (two steps), >99% ee. (xii) 58; LHMDS, THF, −78 °C; NCCO_2_Me, −78 °C to rt.; K_3_PO_4_, DMF, rt; 69% (2 steps); TBHP, Triton B, MeCN/benzene, 62%; (xiii) TFAA, Et_3_N, CH_2_Cl_2_, 0 °C; evaporation; AcOH, Mg(ClO_4_)_2_, benzene, 40 °C; (xiv) NaBH(OCOCF_3_)_3_, THF, rt; TFA; 5-hexenoyl chloride, Et_3_N, 0 °C, 80% (2 steps); (xv) LiAlH_4_, AlCl_3_, Et_2_O, −20 to −10 °C, 93%; (xvi) IBX, t-BuOH, 70 °C; PhSH, Cs_2_CO_3_, MeCN, 50 °C; NaBH(OCOCF_3_)_3_, THF, rt, 89% (2 steps); (xvii) 69 (1.0 equiv.), PMPOH, CH_2_Cl_2_ (1 mM), 41%; (xviii) TBAF, THF, 50 °C; evaporation; H_2_, Lindlar’s catalyst, quinoline, MeOH, 84%; Dess–Martin periodinane, CH_2_Cl_2_, rt, 87%; (xix) tryptamine-TFA, CH_2_Cl_2_, MS3A, rt; TFA, CH_2_Cl_2_; DDQ, CH_2_Cl_2_/benzene, rt, 75% (3 steps).

Fukuyama and colleagues [91,92] achieved the first enantioselective total synthesis (shown in Figure 5). A key strategic element was a stereospecific [3,3]-sigmatropic rearrangement of an allylic cyanate, serving as a “traceless” method through which to install the crucial nitrogen atom of the B-ring with precise stereochemistry. This route confirmed the robustness of RCM for macrocycle formation and stood as a masterpiece of stereocontrolled design.

##### The Most Efficient Synthesis to Date (Dixon Route, 2012)

**Figure 6:**
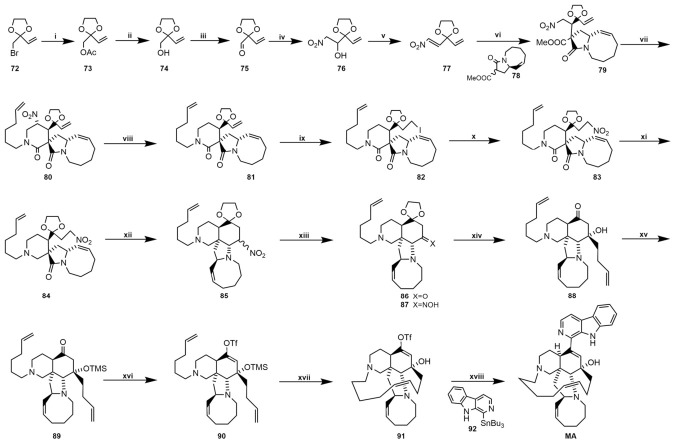
Total synthesis of MA by Dixon and coworkers. Reagents: (i) KOAc, Aliquat 336, 120 °C; (ii) K_2_CO_3_, MeOH, rt, 49% (84% brs, two steps); (iii) COCl_2_, DMSO, Et_3_N, CH_2_Cl_2_, −78 °C to rt; (iv) CH_3_NO_2_, EtOH, 0 °C, 90% (over two steps); (v) MsCl, Et_3_N, CH_2_Cl_2_, −15 °C to rt, 90%; (vi) 78, KHMDS, 18-crown-6, −94 °C, THF, 65%; (vii) HCHO, hex-5-en-1-amine, refluxing MeOH, 88%; (viii) AIBN, Bu_3_SnH, toluene, reflux, 77%; (ix) TMSCl, KI, 4AMS, MeCN, rt, 81%; (x) AgNO_2_, Et_2_O, rt, 63%; (xi) DIBAL, toluene, −78 to −20 °C, 74%; (xii) Ti(OiPr)_4_, Ph_2_SiH_2_, hexane, 0 °C, 81% (dr 83:17); (xiii) TiCl_3_, THF, water, 56% of 80, 21% of 81; (xiv) 3-butenylmagnesium bromide, THF, CeCl_3_, 0 °C; 0.5 h then HCl, 40 h, rt, 91%; (xv) TMSOTf, Et_3_N, Et_2_O, 72%; (xvi) Commins’ reagent, KHMDS, THF, −78 °C, 90%; (xvii) Grubbs first-generation catalyst (20 mol%), CH_2_Cl_2_, reflux, 73%; (xviii) Pd(PPh_3_)_4_ (12 mol%), 92, DMF, 60 °C, 52%.

Dixon’s synthesis [91,93] represents the current apex of efficiency, with the shortest linear sequence (18 steps, as shown in Figure 6). It employs a highly convergent strategy, coupling two readily prepared fragments. Its brilliance lies in the extensive use of tandem reactions, most notably a nitro-Mannich/lactamization and a reductive nitro-Mannich cascade, which build the core piperidine and bridged systems with exceptional step economy. The synthesis culminates in late-stage palladium-catalyzed Stille coupling to install the β-carboline unit, making this route exceptionally adaptable for preparing diverse manzamine analogues.

#### 2.4.4. Formal Total Syntheses via Furan–Iminium Cation Cyclization (FIC) (Nishida Route, 2016)

**Figure 7:**
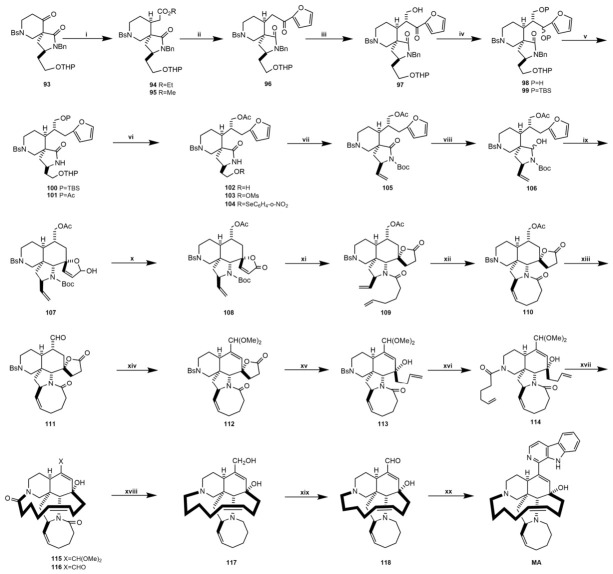
Total synthesis of MA by Nishida and coworkers. Reagents: (i) Ph_3_P=CHCO_2_Et, toluene, reflux, 80%; H_2_, cat. PtO_2_, MeOH-H_2_O(5:1); HNMe(OMe), *i*-PrMgCl, THF, −20 °C; (ii) 2-furyl lithium, THF, −78 °C, 84% (3 steps); (iii) HCHO, DBU, 74%; (iv) NaBH_4_, MeOH, 0 °C; TBSOTf, 2,6-lutidine, CH_2_Cl_2_, −78 °C; (v) Li, NH_3_, −40 °C; BsCl, NaHCO_3_, EtOAc-H_2_O, 61%; TBAF, THF; Ac_2_O, pyridine; (vi) *p*-TsOH, *i*-PrOH-CH_2_Cl_2_, 88% (3steps); MsCl, pyridine; o-NO_2_PhSeCN, NaBH_4_, DMF; (vii) 30% H_2_O_2_aq., THF, 64% (3 steps); (viii) (Boc)_2_O, Et_3_N, cat. DMAP, THF, 98%; LiBH_4_, THF; Ac_2_O, pyridine; (ix) p-TsOH, ace-tone-H_2_O; (x) IBX, DMSO, 50 °C, 70% (4 steps); (xi) NaBH_4_, cat. NiCl_2,_ MeOH; TFA, CH_2_Cl_2_, 0 °C to rt.; 5-hexenoyl chloride, DMAP, Et_3_N, CH_2_Cl_2_, 83% (3 steps); (xii) Grubbs’ second (10 mol%), CH_2_Cl_2_, reflux, 90%; (xiii) KCN, MeOH-CH_2_Cl_2_; Dess-Martin periodinane, CH_2_Cl_2_, 0 °C, 91% (2 steps); (xiv) TMSBr, Et_3_N, CH_2_Cl_2_, rt; Pd(OAc)_2_, CH_3_CN; HC(OMe)_3_, p-TsOH-H_2_O, MeOH, rt, 78% (3 steps); (xv) DIBAL, CH_2_Cl_2_, −78 °C; Ph_3_PCH_3_Br, KHMDS, THF, 0 °C to rt, 65% (2 steps); (xvi) Na, naphthalene, DME, −65 °C; 5-hexenoyl chloride, DMAP, Et_3_N, CH_2_Cl_2_, 88% (2 steps); (xvii) Grubbs 1st (20 mol%), CH_2_Cl_2_ (degassed), reflux; 1N HCl, EtOAc, 63% (2 steps); (xviii) DIBAL, CH_2_Cl_2_, −78 °C to rt.; (xix) Dess-Martin periodinane, CH_2_Cl_2_, 0 °C to rt. 21% (2 steps). (xx) tryptamine, toluene, TFA; DDQ, EtOH.

Formal total synthesis (shown in Figure 7) of MA, as reported by Nishida et al. [91,94], is achieved through the strategic construction of its advanced biogenetic precursor, ircinal A. The synthesis is characterized by a convergent and stereocontrolled route centered on a key furan–iminium cation cyclization (FIC). This efficient, stereoselective 6-endo-trig cyclization simultaneously forms a six-membered ring and establishes a tetrasubstituted stereocenter, providing rapid access to the manzamine core before elaboration via RCM, highlighting the FIC reaction as a powerful method for the rapid and stereocontrolled assembly of complex polycyclic alkaloid frameworks.

### 2.5. Derivatives and Analogues

As a complex β-carboline alkaloid with a unique polycyclic structure, the chemical diversity of MA is extremely extensive, and MA has served as a privileged scaffold for the discovery of numerous derivatives and analogues with diverse biological activities [22]. These compounds can be categorized as follows and their structural characteristics, representative drugs, and primary actions are summarized in Table 6.

#### 2.5.1. Natural Analogues

**Figure 8:**
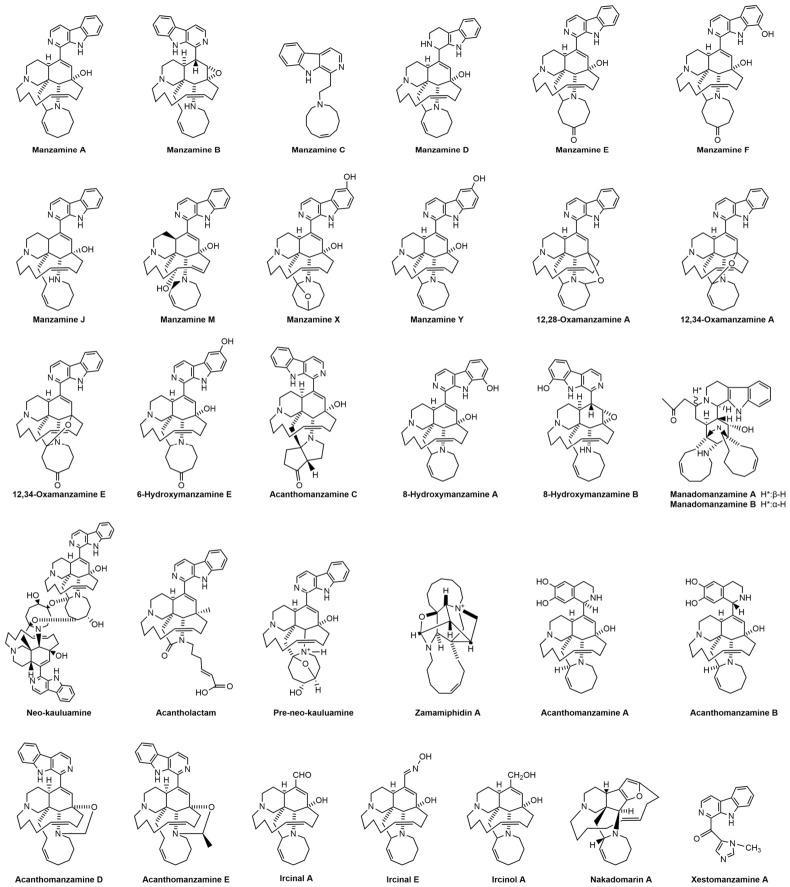
The chemical structures of MA and its natural analogues.

This category encompasses all manzamine-type alkaloids obtained from natural sources, including both the sponge host and its symbiotic microorganisms. Sponge-derived analogues refer to compounds directly isolated from marine sponges (primarily genera *Acanthella*, *Amphimedon*, etc.), which together with MA constitute the manzamine alkaloid family, including manzamine B, C, E, F, X, Y, ircinals, etc. [75]. These compounds mainly reflect the oxidation, rearrangement and modification changes in the biosynthetic pathways and serve as a reservoir for lead compounds’ discovery [95]. They exhibit a range of significant pharmacological activities that establish a foundation for the manzamine alkaloid family’s therapeutic potential. Their activities, particularly antiparasitic, antiviral, and anticancer, form the basis for both semisynthetic modification and synthetic analogue development. Recent studies on the symbiotic microbes (e.g., *Micromonospora* sp. M42) of manzamine-producing sponges have revealed related alkaloids, such as manadomanzamines, 8-hydroxymanzamine A, etc. [57]. These compounds typically share the β-carboline core and exhibit similar bioactivity profiles, including antimicrobial, anti-inflammatory, and neuroprotective effects, suggesting a broader chemical and therapeutic space within this alkaloid class [96]. The chemical structures of MA and its representative natural analogues are shown in Figure 8.

**Table 6:** The derivatives and analogues of MA.

Category	Types	Structural Characteristics	Representative Members	Primary Actions
Natural Analogues	Sponge-derived analogues [75,95]	These feature the core manzamine scaffold with variations such as hydroxylation, peroxidation, or ring rearrangement	Manzamine B, C, E, F, X, Y, Ircinals	They exhibit core antimalarial, anticancer, and antiviral activities, with potencies varying based on structural modifications.
	Symbiont-derived analogues [57]	These share the β-carboline core	Manadomanzamines, 8-hydroxymanzamine A	They exhibit similar bioactivity profiles, showing antimicrobial, anti-inflammatory, and neuroprotective potentials and highlighting a broader ecological and therapeutic role.
Semi-Synthetic Derivatives	Functional group modifications [23,60,74,84,95,97]	These often target the β-carboline core or the complex polycyclic ring system	2-N-Methylmanzamine A, 8-Acetoxymanzamine A, 8,12-Diacetoxymanzamine A, 8-Methoxymanzamine A, 12,13-Dehydromanzamine A	They generally aim to retain or enhance the parent compound’s bioactivity profile (e.g., antiparasitic, anticancer), improve solubility (e.g., salts), metabolic stability, reduce toxicity or create prodrugs.
Simplified Synthetic Analogues	β-Carboline-focused analogues [25]	These retain the β-carboline core but with simplified or replaced polycyclic ring systems	compound **125**, compound **126**	They are designed for more feasible synthesis while mimicking MA’s key interactions. These analogues validate the β-carboline moiety as crucial for antimalarial and cytotoxic/anticancer activities, facilitating SAR studies and lead optimization.
Mechanism-Oriented Derivatives	Kinase inhibitor probes [25,77]	They are developed based on MA’s identified off-target kinase inhibition	6-methoxymanzamine A, Methyl manzamine A-3-carboxylate, 9N-butylmanzamine A	They are tools for studying diseases like Alzheimer’s (GSK-3β, CDK5) and cancer (RSK1), representing a repurposing of the scaffold for targeted therapy.

#### 2.5.2. Semi-Synthetic Derivatives

**Figure 9:**
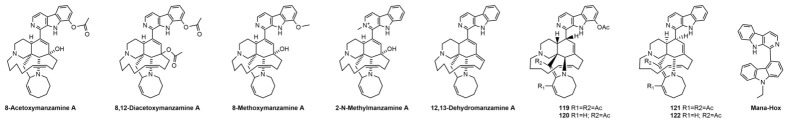
The chemical structures of MA’s representative semi-synthetic derivatives.

To improve activity, selectivity, or pharmacokinetic properties [97], some derivatives (e.g., Manzamine A hydrochloride, ester/ether derivatives, reduction products, oxime and hydrazone derivatives, etc.) [60] have been synthesized by chemical modification of natural MA, and structural modifications often target the β-carboline core or the complex polycyclic ring system [95,97]. The chemical structure of a typical compound is shown in Figure 9. They represent an attempt at rational medicinal chemistry, aiming to overcome the difficulties of synthesizing natural products, optimize their biological properties, and elucidate the essential pharmacophores that enable their functions. Semisynthetic derivatives primarily exhibit potent antiparasitic activity, especially against Plasmodium falciparum (malaria), with some showing improved efficacy or reduced cytotoxicity compared to the parent compound [23]. Significant antiviral activity against pathogens like HIV-1 and herpes simplex virus (HSV-1) has also been documented [74]. Furthermore, several derivatives demonstrate anticancer properties through mechanisms including cytotoxicity, induction of apoptosis, cell cycle arrest, and specific inhibition of oncogenic targets like the SIX1 protein [84].

#### 2.5.3. Simplified Synthetic Analogues

**Figure 10:**
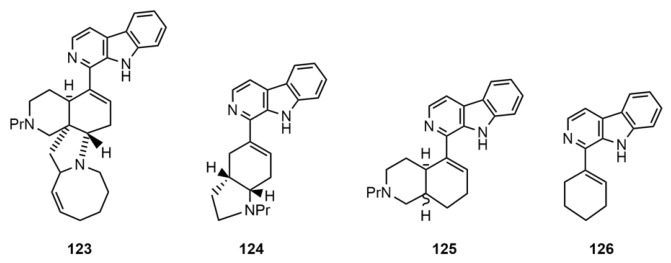
The chemical structures of MA’s representative simplified synthetic analogues.

To address the synthetic complexity of the full manzamine scaffold, researchers have developed simpler analogues focusing on the β-carboline or other key pharmacophores. These compounds (**125**, **126**, etc.) aim to retain or enhance bioactivity while improving synthetic accessibility [25], and the chemical structures of representative compounds are shown in Figure 10. Many of these simplified analogues maintain antimalarial and anticancer activities, validating the importance of core structural motifs for biological function.

#### 2.5.4. Mechanism-Oriented Derivatives

**Figure 11:**
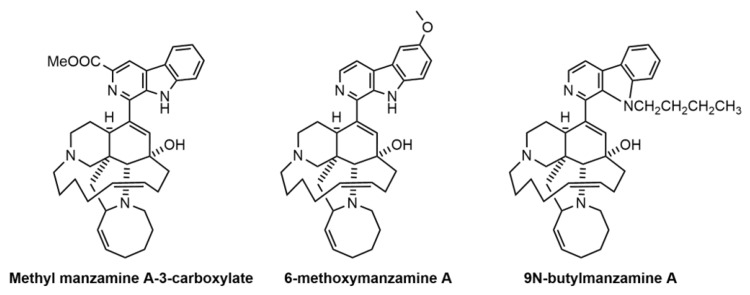
The chemical structures of MA’s representative mechanism-oriented derivatives.

Some research has focused on derivatives (6-methoxymanzamine A, Methyl manzamine A-3-carboxylate, 9N-butylmanzamine A, etc.) designed to probe specific mechanisms of action. For instance, analogues have been evaluated as inhibitors of glycogen synthase kinase-3β (GSK-3β), cyclin-dependent kinase 5 (CDK5), and ribosomal S6 kinase 1 (RSK1), linking manzamine’s scaffold to kinase inhibition relevant to cancer, Alzheimer’s disease, and other conditions [25,77]. The chemical structures of representative compounds are shown in Figure 11.

Collectively, the scaffold of MA has inspired a diverse chemical arsenal. Natural derivatives and semi-synthetic analogues form the foundation, primarily explored for their potent anti-infective and anticancer properties. Simplified synthetic analogues make this pharmacophore more accessible for drug development. Microbial biosynthetic analogues reveal the ecological origin and expand the structural diversity for new biological functions. Finally, mechanism-oriented derivatives represent a modern, targeted approach, refining the scaffold into probes and leads for specific diseases like neurodegeneration and oncology. Collectively, these categories underscore MA’s significance as a versatile lead structure in medicinal chemistry and drug discovery.

## 3. Discussion

Manzamine-A (MA), a macrocyclic alkaloid, exhibits remarkable multi-target pharmacological activities, especially showing unique potential in anticancer, antimalarial, and neuroprotective applications [84]. Its complex acridine–carbazole–imidazoline polycyclic structure underpins its distinctive bioactivity and mechanisms of action. However, despite these promising pharmacological effects, the translational development of MA faces an immediate and formidable challenge: sustainable supply [98]. For decades, MA was considered a definitive nature product of marine sponges [99]. The natural product’s intricate architecture, while responsible for its bioactivity, also renders its extraction from marine sponges ecologically unsustainable and low-yielding [57]. This bottleneck has necessitated a deeper exploration of its origin and the development of alternative production strategies. However, a paradigm shift occurred with the discovery that its biosynthesis is not carried out by the sponge itself but by a symbiotic bacterium, specifically *Micromonospora* sp. This revelation redefined the compound’s ecological and evolutionary context and opened new avenues for its production through fermentation. This finding serves as the cornerstone for understanding the molecule’s natural history.

Additionally, this supply bottleneck has also propelled significant efforts in MA synthesis. The total synthesis of MA is a historic narrative of synthetic organic chemistry’s evolution. The field has transitioned from linear, step-intensive proofs of concept (Winkler, Martin) to enantioselective marvels (Fukuyama) and ultimately to convergent, tandem reaction-driven models of efficiency (Dixon). Contemporary efforts focus on novel disconnections (Nishida) and biomimetic inspiration (Kerr) [100], vividly illustrating the paradigm shift in complex molecule synthesis from “can it be made?” to “how can it be made more efficiently and biomimetrically?”. Future directions are increasingly driven by medicinal chemistry objectives. The paradigm exemplified by the Dixon synthesis—generating a versatile, late-stage intermediate for rapid diversification—is poised to dominate, enabling efficient exploration of SAR and the pharmaceutical potential of this remarkable alkaloid class. The pursuit of a truly biomimetic, one-step cyclization from an advanced precursor remains an enticing and unresolved challenge for the future.

While total synthesis establishes chemical access, it concurrently highlights another layer of complexity: the innate physicochemical properties of the natural product often conflict with drug-like requirements. Specifically, MA exhibits suboptimal pharmacokinetic (PK) characteristics. Its poor solubility, low oral bioavailability, unclear distribution and clearance profile, metabolic instability, and potential drug interaction risks also significantly limit direct clinical translation [25]. Future research priorities might include the following: (1) Conducting systematic preclinical ADME studies, utilizing radiolabeled MA to fully quantify its absorption, distribution, metabolism, and excretion parameters in multiple animal models; (2) Elucidating its metabolic fate by employing sensitive techniques like LC-MS/MS to identify its major metabolites and the involved CYP enzymes; (3) Optimizing formulation strategies by developing nano-formulations (e.g., liposomes, polymeric micelles), prodrugs, or co-crystals to significantly improve its solubility, stability, and oral absorption; and (4) Carrying out structure-based PK optimization by designing next-generation analogues that retain the core pharmacophore through structural modification to reduce affinity for P-gp or susceptibility to specific CYP enzymes, thereby yielding an improved PK profile. Therefore, merely replicating the natural structure is insufficient; strategic structural modification becomes imperative.

This necessity directly bridges chemical synthesis and medicinal chemistry optimization. Leveraging insights into its PK deficiencies and pharmacological targets, a rich array of semi-synthetic derivatives and analogues have been designed and synthesized by using the core scaffold of MA as a versatile template to streamline synthetic access through retrosynthetic analysis and to enhance drug-like properties via rational structural modification. The feasibility of this optimization strategy is evidenced by structure–activity relationship (SAR) studies, which have yielded derivatives with improved PK profiles while maintaining core biological activity [73]. Discoveries of new analogues from natural sources further provide valuable structural blueprints for rational design. This expanding chemical library exhibits predominant and promising activities against malaria, cancer, and viruses, with emerging roles in modulating specific kinases and other cellular pathways. Ongoing research into their mechanisms of action continues to unveil their potential as multifaceted lead compounds. Future directions will logically extend from this established framework, focusing on further structural optimization for selectivity and PK properties, as well as the exploration of newly identified effects on processes such as bone homeostasis.

In summary, future research could focus on the following: (1) structural optimization through developing semi-synthetic or computationally designed derivatives with enhanced efficacy and reduced toxicity; (2) mechanistic elucidation by utilizing omics technologies to clarify precise molecular targets, such as GSK-3β inhibition and autophagy modulation; (3) combination therapy exploring synergies with existing drugs (e.g., artemisinin for malaria and targeted anticancer agents); and (4) scalable production leveraging synthetic biology and enzyme engineering for efficient and sustainable biosynthesis. Only through interdisciplinary efforts to address sourcing, toxicity, and druggability challenges can MA evolve from a marine natural product into a clinically viable therapeutic agent.

## Figures and Tables

**Table 1:** Antimalarial activities and antileishmanial activities of MA.

Drugs	Antimalarial Activities (IC50)								Antileishmanial Activities (IC50)		
	*Plasmodium falciparum* [57,63,64,66] *(a = Chloroquine-Sensitive Cell Lines; b = Chloroquine-Resistant Cell Lines)*								*Lesishmania donovani* [66]		
	*-*	*^a^ D6*				*^b^ W2*					
	nM	nM	μM	ng/mL	μg/mL	nM	μM	ng/mL	nM	mg/mL	µg/mL
MA	25	8.0	0.017	20.8 ± 0.75	0.0045	11	0.020	25.8 ± 7.5 /8.0	6.2	11.15 ± 1.15	0.9
chloroquine	53	50	0.013	10 ± 4.7 /15.5	-	484	0.135	107 ± 17.5 /170	-	-	-
artemisinin	-	46	0.0063	4.7 ± 0.3 /10	-	28	0.0045	3.8 ± 0.8 /6.3	-	-	-
pentamidine	-	-	-	-	-	-	-	-	47	1.65 ± 0.05	2.1

**Table 2:** Antibacterial activities of MA.

Drugs	Antibacterial Activities (IC50)							
	*Staphylococcus aureus* [68]	*Mycobacterium intracellular* [57,63,66,69]				*Mycobacterium tuberculosis* [66,68]	*Methicillin-Resistant S. aureus (MRSA)* [66,68]	
	µg/mL	nM	μM		µg/mL	μg/mL	μM	µg/mL
MA	0.5	0.36	10	0.640	0.36 ± 0.01 /0.35	1.5	0.18	1.80 ± 0.03 /0.7
ciprofloxacin	0.10	0.18	1.1	1.056	0.48 ± 0.01 /0.25	-	0.3	0.13 ± 0.02 /0.10
rifampicin	-	-	-			0.5	-	-

**Table 3:** Antifungal activities of MA.

Drugs	Antifungal Activities (IC50)					
	*Cryptococcus neoformans* [63,64,66,72]			*Candida albicans* [63,64,66]		
	μM		µg/mL	μM		µg/mL
MA	2.7	1.848	7.25 ± 1.08 /3.0	16.4	3.656	Inactive up to 20
amphotericin B	0.8	0.920 /2.705	1.14 ± 0.07 /0.15	0.3	0.487 /1.352	0.32 ± 0.04

**Table 4:** Antiviral activities of MA.

Drugs	Antiviral Activities (IC50/EC50)	
	*HSV-1* [74]	*HIV-1* [24]
	µM	μM
MA	1	4.2
acyclovir	50	-
zidovudine	-	0.004

**Table 5:** Anti-tumor activities of MA.

Tumor Types	Tumor Cells	Anti-Tumor Mechanisms of MA
Pancreatic cancer [28,80,81]	AsPC-1, PANC-1, BxPC-3, MIA PaCa-2	MA decreases single cell formation, abrogates cell migration and restores the susceptibility to TRAIL-induced apoptosis
		MA inhibits vacuolar-ATPase, blocks autophagosome turnover, and impairs autophagy
Colorectal cancer [27,80,82,83,84]	HCT116, HT-29, DLD-1	MA induces cell cycle arrest at G_0_/G_1_ phase through p53/p21/p27 signalling inhibition, triggers a caspase-dependent apoptotic cell death, and prevents epithelial-mesenchymal transition (EMT) process
Cervical cancer [29]	C33A, HeLa, SiHa, CaSki	MA decreases the levels of the oncoprotein SIX1, inhibits the kinase activity of RSK1 and RSK2, and causes cell cycle arrest at the G1/S phase
Breast cancer [30,85]	MCF-7, MDA-MB-231	MA induces secretory autophagy through the RIP1/AKT/mTOR pathway
Prostate cancer [86]	LNCaP, 22Rv1, PC3, DU145	MA targets transcription factor E2F8 to block the transcription of androgen receptor (AR) and its splice variant AR-V7, a key driver of therapy-resistant prostate cancer
Glioblastoma [87]	U373, U87	MA induces apoptosis by inhibiting GSK3β activation, which downregulates oncogenic splicing factors (SRSF1, hnRNPA1) and anti-apoptotic proteins (Survivin, BCL2) while restoring tumor suppressor Anxa7 expression
Uterine fibroids [88]	ELT-3, HUtSMC	MA inhibits cell proliferation and extracellular matrix deposition by targeting SOAT/β-catenin to induce oxidative stress and ER stress

## Data Availability

The original data are available from the corresponding author on request.
